# A Rapid and Sensitive Assay for the Detection of Benzylpenicillin (PenG) in Milk

**DOI:** 10.1371/journal.pone.0132396

**Published:** 2015-07-13

**Authors:** Anna Pennacchio, Antonio Varriale, Maria Grazia Esposito, Andrea Scala, Vincenzo Manuel Marzullo, Maria Staiano, Sabato D’Auria

**Affiliations:** 1 Laboratory for Molecular Sensing, IBP-CNR, Naples, Italy; 2 Institute of Food Science, ISA- CNR, Avellino, Italy; Vrije Universiteit Brussel, BELGIUM

## Abstract

Antibiotics, such as benzyl-penicillin (PenG) and cephalosporin, are the most common compounds used in animal therapy. Their massive and illegal use in animal therapy and prophylaxis inevitably causes the presence of traces in foods of animal origin (milk and meat), which creates several problems for human health. With the aim to prevent the negative impact of β-lactam and, in particular, PenG residues present in the milk on customer health, many countries have established maximum residue limits (MRLs). To cope with this problem here, we propose an effective alternative, compared to the analytical methods actually employed, to quantify the presence of penicillin G using the surface plasmon resonance (SPR) method. In particular, the PenG molecule was conjugated to a protein carrier to immunize a rabbit and produce polyclonal antibodies (anti-PenG). The produced antibodies were used as molecular recognition elements for the design of a competitive immune-assay for the detection of PenG by SPR experiments. The detection limit of the developed assay was found to be 8.0 pM, a value much lower than the MRL of the EU regulation limit that is fixed at 12 nM. Thus, our results clearly show that this system could be successfully suitable for the accurate and easy determination of PenG.

## Introduction

Antibiotics are the main class of compound widely used to prevent and/or treat animal diseases, such as mastitis. In particular, the most commonly used compounds are penicillin G and cephalosporin, which belong to the β-lactams family [1]. Penicillin G is the antimicrobial more frequently sought through the Food Animal Residues Avoidance Databank (FARAD) and is one of the most commonly detected drug residues in animal tissue and milk [2]. The presence of antibiotic residues in milk products is most likely due to their injudicious use in the treatment of animal infections [3]. In fact, antibiotics are used as food additives, and their massive and/or illegal use inevitably causes the presence of traces in foods of animal origin (milk and meat), creating several problems for human health [4]. The presence of drug residues in milk and in other daily supplies and products is of a public health interest and is perceived by consumers as undesirable [5,6].

The main effects of human exposition to this class of compounds are allergic reactions, bacterial resistance to β-lactams and long-term toxic effects due to their potential carcinogenicity, mutagenicity and teratogenicity, as described in a report by Epstein [7]. Therefore, antibiotic contamination in food is a public health concern.

With the aim to prevent the negative impact of β-lactams, and in particular of PenG residues present in milk, on customer health, many countries have established maximum residue limits (MRLs). European Union (EU) Regulation 508/1999 has established the MRLs in milk and in meat for some antibiotics: for benzyl-penicillin in milk (penicillin G), the MRL is 12 nM (Codex Alimentarius Commission, Maximum Residue Limits for Veterinary Drugs in Foods Updated as at the 34th Session of the Codex Alimentarius Commission 2011).

Actually, three different methods for the detection of antibiotic residues are primarily applied: microbiological assays, analytical methods (HPLC, GC, etc.) and immunoassay methods [8–13].

All of these technologies present different limitations that make it difficult to extend the detection of PenG outside of the laboratory. A rapid, specific and sensitive assay that is usable in the field and, in particular, in all steps of milk production, such as in the cattle shed, in milk collection and in the consumer’s home, is needed. This assay would allow the ability to control all phases of milk production with the consequent reduction of human exposure to antibiotic contamination. Biosensor application, however, offers a valuable alternative detection method to cope with the necessity to enable a fast, easy and specific approach for food matrices analysis. In recent years, different biosensors, including hybrid biosensors [14], electrochemical biosensors [15] and surface plasmon resonance imaging/surface plasmon resonance (SPR) immune-sensors [16–18], have been developed for PenG detection.

SPR-based biosensors have been widely used as tools for characterizing and quantifying bio-molecular interactions as well as for detection of analytes associated with medical diagnostics, environmental monitoring, food safety and homeland security. The targeted analytes in food safety field include different molecules as reported from Homola [19]. SPR-based detection is widely used because it is an analytical technique easy to use and furthermore it requires a simple and fast sample preparation as well as a reduced assay time [19].

Recently, our laboratory contributed to the knowledge about this topic, developing a SPR assay for the detection of two different analytes, patulin [20] (food toxin produced by different species of fungi) and ephedrine [21] (drug precursor of amphetamine). Both these assays are based on the use of specific antibody molecules produced against the selected analytes, enabling the rapid, sensitive and specific detection of selected analytes in matrices of interest.

In this work, we present a novel sensing approach to quantify the presence of penicillin G using a SPR technique with the SensiQ discovery portable instrument. The assay is based on the use of the *ad hoc* synthesized penicillin G-GlnBP (PenG-GlnBP) conjugate, immobilized on the gold surface of a SensiQ chip, and specific antibodies generated against PenG. A competitive immunoassay based on the use of produced polyclonal mono-specific antibodies was performed to directly detect penicillin G in milk solutions.

## Materials and Methods

### Reagents

All reagents were purchased at the highest purity that was commercially available. 1-[3-(Dimethylamino)-propyl]-3-ethylcarbodiimide (EDC), bovine serum albumin (BSA; fraction V), ovalbumin (OVA; grade V) and the PURE1A Protein A Antibody Purification Kit were purchased from Sigma. Goat polyclonal anti-rabbit IgG-HRP conjugate (secondary antibody) was from Abcam. Affinity resin EAH Sepharose 4B was purchased from Amersham Biosciences. Nitrocellulose transfer membrane Protran from Schleicher & Schuell and ECL detection reagents from Amersham Biosciences were used in the dot blot and western blot experiments. Microplates (96-well), LockWell MaxiSorp from Nunc, 3,5-tetramethylbenzidine (TMB) enzyme substrate from Sigma, and a microplate reader, Multiskan EX from Thermo, were used for the ELISA experiments. UV measurements were carried out on a Varian Cary 50 Bio spectrophotometer. Antibodies against benzyl-penicillin (anti-PenG) were produced and purchased from COVALAB S.A.S. (Villeurbanne, France). HBS-EP buffer was purchased from Biacore (GE Healthcare). The milk was produced from Granarolo (Granarolo S.p.a. Italy).

### Synthesis of the BSA Penicillin G conjugate (PenG-BSA)

The penicillin G-BSA conjugate (PenG-BSA) was prepared according to Levine [22], with slight modifications. Briefly, BSA (10 mg) was dissolved in 2 mL of sodium carbonate buffer (100 mM, pH 10.5). Penicillin G (5.5 mg; 100-fold molar excess) was added, and the reaction mixture was incubated for 16 h at 4°C. Finally, an extensive dialysis against potassium phosphate buffer (20 mM, pH 7.2; 0.5 L) was performed for 3 days with daily buffer changes, and the conjugate concentration was determined spectrophotometrically at 278 nm.

### Antibody production and purification

**Fig 1:**
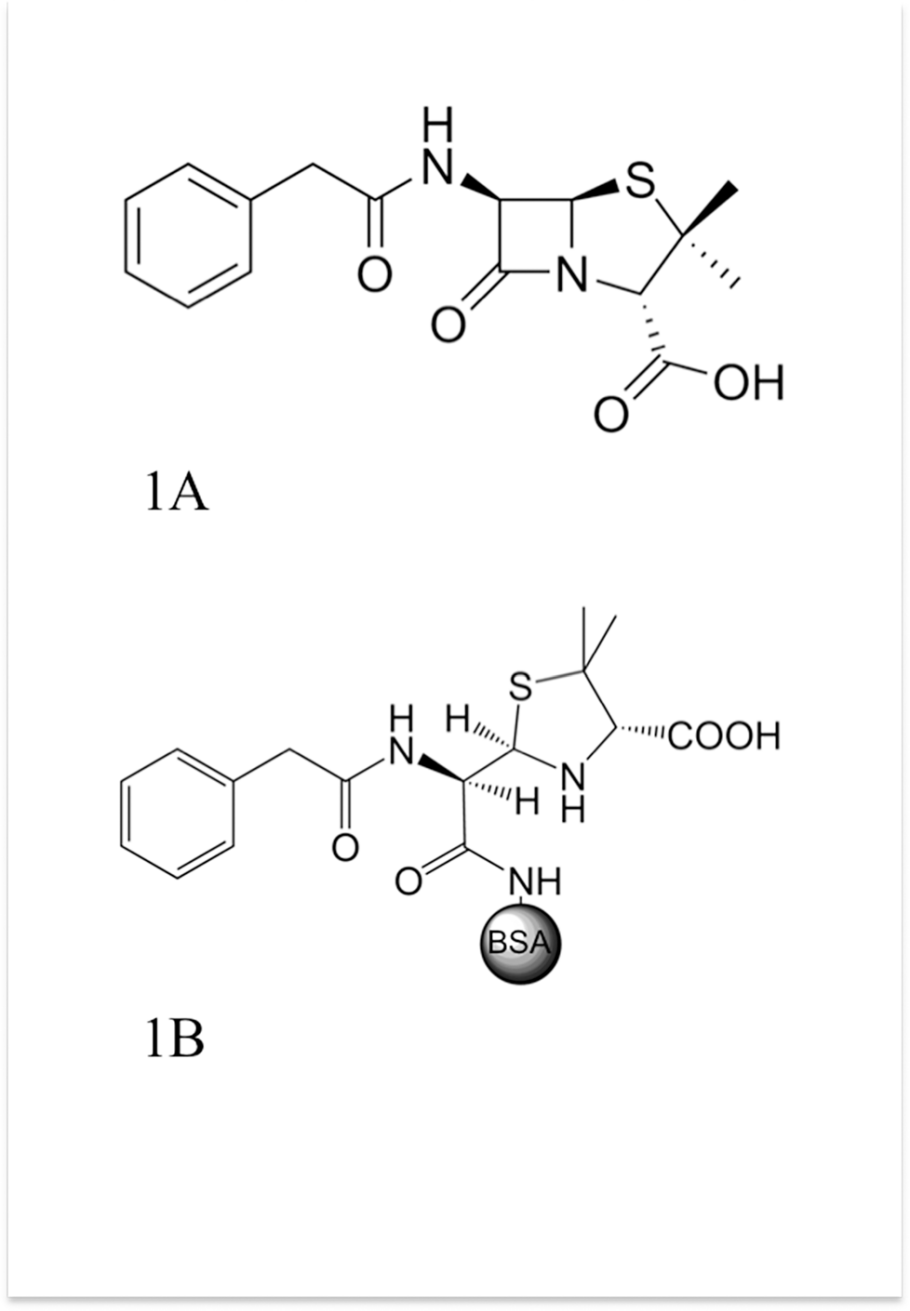
PenG molecule and PenG-BSA conjugate. Penicillin G structure (A) and schematic representation of the PenG-BSA conjugate (B).

The antibodies used in the work were produced and purchased from COVALAB SAS France (COVALAB, Villeurbanne, France). The company used as an antigen the conjugate reported in Fig 1B. From the serum provided from COVALAB, rabbit serum (2 ml) was loaded on a Protein A column, and the IgG fraction was eluted with glycine buffer (0.1 M) at pH 2.8 and immediately buffered in Tris/HCl (1.0 M) at pH 9.0. The IgG fraction concentration was calculated by absorbance at λ = 278 nm, and its purity was checked by sodium dodecyl sulfate-polyacrylamide gel electrophoresis (SDS-PAGE).

### Affinity column preparation of Penicillin G–EAH Sepharose 4B and antibody purification

The affinity column was achieved by conjugating PenG to EAH Sepharose 4B and was prepared according to De Champdore [23] and Pennacchio [20].

An aliquot of total IgG (2.0 mL), previously purified, was loaded on the affinity chromatography column PenG conjugated-EAH Sepharose 4B and mono-specific antibodies were obtained. In particular, unspecific antibodies were washed out with three high-salt different buffer solutions: (1) PBS (0.01 M), NaCl (0.1 M), pH 7.0 (20 mL); (2) PBS (0.01 M), NaCl (0.5 M), pH 7.0 (20 mL); and (3) PBS (0.01 M), NaCl (1.0 M), pH 7.0 (20 mL) [23].

Mono-specific antibodies anti-PenG were eluted with glycine (0.1 M), pH 2.8 (2.5 mL). They were concentrated and dialyzed against a solution containing PBS (0.1 M) and NaCl (0.1 M), pH 7.4. The concentrations of the antibodies were determined by absorbance measurements at 278 nm. The sample purity was evaluated by SDS-PAGE analysis.

### Synthesis of GlnBP Conjugates (PenG-GlnBP)

PenG was conjugate to the glutamine-binding protein (GlnBP) from *E*. *coli*. GlnBP used for conjugation was produced according to Staiano et al [24], and the PenG conjugate to GlnBP (PenG-GlnBP) was prepared according to Levine [22].

### Antibody Titration

The antibody titer was determined by an indirect ELISA assay following the general procedure reported by Kuck [25]. In brief, PenG-GlnBP was dissolved in PBS (0.1 M), pH 7.4 and was deposited on coat 96-well micro-plates surface in a range of concentrations from 1.1 ng/mL to 1.7 ng/mL and BSA dissolved in the same buffer was used as control sample.

All the wells were incubated overnight at 4°C, washed three-times with PBS buffer (0.1 M) containing 0.05% Tween (PBS-T), pH 7.4, and blocked by incubation for 2.0 hours at room temperature with PBS-T buffer containing BSA (1%).

The wells were washed several times with PBS-T after each step, incubated with anti-PenG antibodies at room temperature for 1.0 hour and, subsequently with horseradish peroxidase-conjugated anti-rabbit IgG antibodies (diluted 1:4000). This solution was incubated for 1.0 hour at room temperature. The enzyme substrate TMB was added, and the color reaction was quenched after 5 min by the addition of 1 M H_2_SO_4_. The absorbance value at 450 nm was measured. Plotting the reciprocal of the antibody dilution against absorbance allowed us to obtain the titer (1/75000) of the anti-PenG antibodies.

### Surface Plasmon Resonance (SPR) experiments

The SPR measurements were carried out on SensiQ discovery instrument by using the CO_2_H_5_ sensor chip. All experiments were carried out three times at a flow rate of 25 μL/min [26] using HBS-EP buffer [26]. The obtained data were de-convoluted using Qdat software (SensiQ Discovery).

### pH Scouting

The procedure used for determining the appropriate immobilization pH is defined as *pH scouting* and was performed with a SensiQ instrument, before immobilizing PenG-GlnBP on the CO_2_H_5_ chip. In this case the sample was diluted in 10 mM sodium acetate at pH 3.5, 4.0, 4.5, 5.0 and 5.5 to a final concentration of 100 ng/mL in each sample. The flow rate was 25 μL/min, and the contact time was 5 min. At the end of the injection, a washing solution (1 M ethanolamine, pH 8.5) was injected to remove any unbound molecules from the chip surface. From the sensorgram analysis, a high immobilization level was found at pH 5.0. This pH value was chosen for the experiments for the immobilization.

### Surface preparation

The carboxy-methylated dextran layer was activated by injecting a 1:1 mixture of 0.05 M N-hydroxysuccinamide (NHS) and 0.2 M N-ethyl-N’-(dimethylaminopropyl) carbodiimide hydrochloride (EDC) in flow cell 2 [27]. The PenG-GlnBP was diluted in 10 mM sodium acetate buffer, pH 5.0, and was immobilized on the flow cell 2 of the chip CO_2_H_5_. The remaining NHS esters were blocked by the injection of a 1.0 M ethanolamine hydrochloride solution (35 μl, pH 8.5). The non-reacted carboxy-methylated dextran layer in flow cell 1 was used as the reference surface.

### Binding measurements

The SPR binding measurements were carried out in the concentration range of 0.0–100 nM of anti- PenG antibodies. The antibody solutions were diluted in HBS-EP buffer (pH 7.4) at the definite concentrations. The binding flow and the time of injection were 25 μL/min and 3 min, respectively. The regeneration process was performed using phosphoric acid (50 mM, pH 3.0) [27]. All SPR measurements were performed three times, and the obtained results were analyzed by Qdat software.

### Competitive assay

A fixed concentration of antibody (100 nM) was incubated with increasing concentrations of PenG (ranging from 0.0 pM to 100 pM). The solutions were fluxed at 25 μl/min on the prepared SPR chip. The time of injection was 3.0 min. After the binding step, a solution of phosphoric acid (50 mM at pH 3.0) was used for the chip regeneration process. Each SPR measurement was performed three times. The obtained results were analyzed by the Qdat software.

## Results and Discussion

The main aim of this work was to develop a very easy and sensitive method to detect traces of PenG in milk based on the SPR technique. PenG is a low molecular weight compound (Fig 1A), and it is too small to stimulate any immunological response. Therefore, to develop the method, we adopted the following strategy: we covalently attached PenG to an immunological carrier as reported in Levine [22] for producing antibodies against PenG. Fig 1B shows the Penicillin G structure covalently bound to BSA (PenG-BSA) in its open-ring form, defined as penicilloyl.

### Purification of antibodies anti-penicillin G and dot blot

Polyclonal antibodies against PenG were produced (COVALB-France) using the PenG-BSA conjugate as an antigen and purified from the serum provided. The IgG fractions were isolated from the serum samples using a Protein A column kit. The homogeneity of the obtained IgG fractions was evaluated by SDS-PAGE (data not shown), and the pure fractions were sequentially pooled, concentrated and dialyzed against PBS buffer. To exclude false reactions due to anti-BSA antibodies present in the rabbit sera, penicillin G was conjugated to the glutamine-binding protein (GlnBP) purified from *E*. *coli*. The GlnBP conjugate was prepared by the same procedures previously used for the BSA conjugate and reported in the Materials and Methods section. The obtained results from the dot blot experiments show that pre-immune serum did not show a response, while the PenG serum of the rabbits gave signals with antigen PenG-GlnBP and with BSA but not with GlnBP (data not shown).

### Preparation of the affinity columns and purification of specific antibodies

To obtain a mono-specific antibody against PenG, an affinity column was prepared by conjugating PenG to an EAH Sepharose-4B resin. The mono-specific antibody was purified, and the specificity against PenG was evaluated by western blot (data not shown). A response to antibody binding was observed only for the conjugate PenG-GlnBP, and a negative response was registered for BSA and GlnBP. This result confirms the specificity of antibodies produced against PenG.

### ELISA Test

To obtain the titer of the purified antibodies, an indirect ELISA test was performed. For this purpose, we coated the micro-plate wells with different concentrations of the antigen PenG-GlnBP, and we tested serially diluted mono-specific antibodies against PenG produced in rabbits. For non-coated wells, no signal was registered as a consequence of incubation with different diluted samples of IgG (data not to show). The obtained results displayed the high quality of the titer of anti-penicillin G antibodies. In fact, it was possible to perform the ELISA test with IgG dilutions from 1 to 100,000. Additionally, a consistent response was detected on coated PenG-GlnBP at a concentration of 1.1 ng/mL (data not to show).

### SPR binding studies

**Fig 2:**
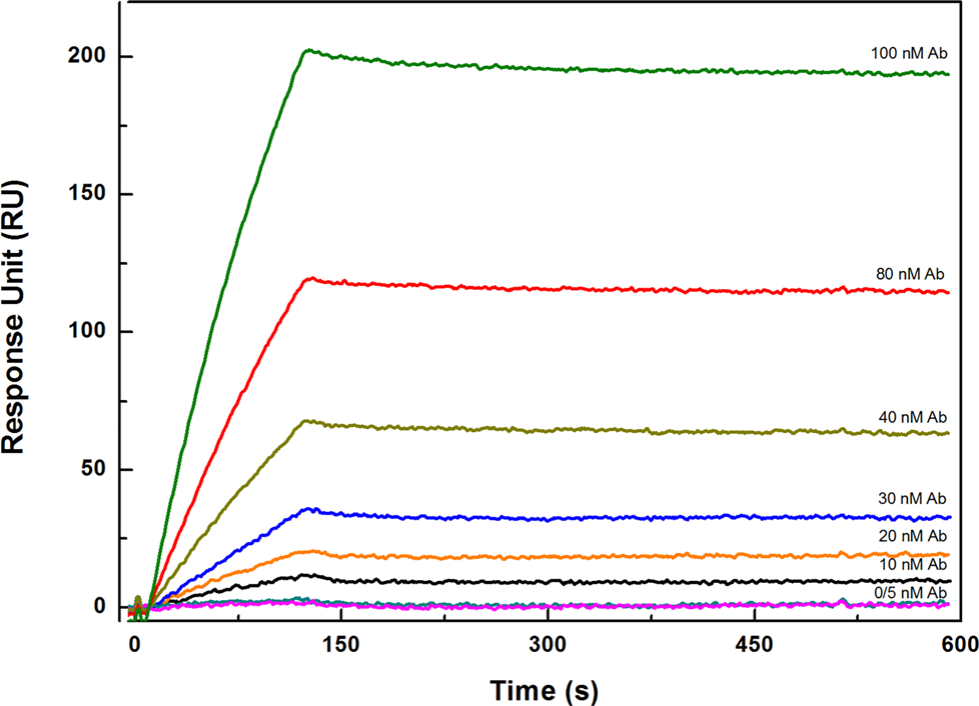
SPR binding experiments. Sensorgram showing the binding of anti-PenG mono-specific antibodies. All measurements were performed in HBS-EP buffer at 25 °C.

The PenG-GnBP was covalently immobilized on the CO_2_H_5_ surface chip by its amino-reactive groups using an amino coupling kit. From the analysis of the pH scouting results (data do not show), we decided to immobilize PenG-GlnBP on the CO_2_H_5_ surface at pH 5.0. To test the sensing system, the binding of polyclonal mono-specific antibodies to the PenG-GlnBP-functionalized CO_2_H_5_ chip was monitored as a function of time. Fig 2 shows the obtained sensorgram, which reported the variation of the Response Unit (RU), as a function of the time, in the absence and presence of a wide range of concentrations of anti-PenG antibodies (0.0 nM to100 nM). From the analysis of the data, it is evident that the RU_max_ signal increases with increasing concentrations of anti-PenG antibodies as a consequence of binding on the surface.

### SPR competitive immunoassay

**Fig 3:**
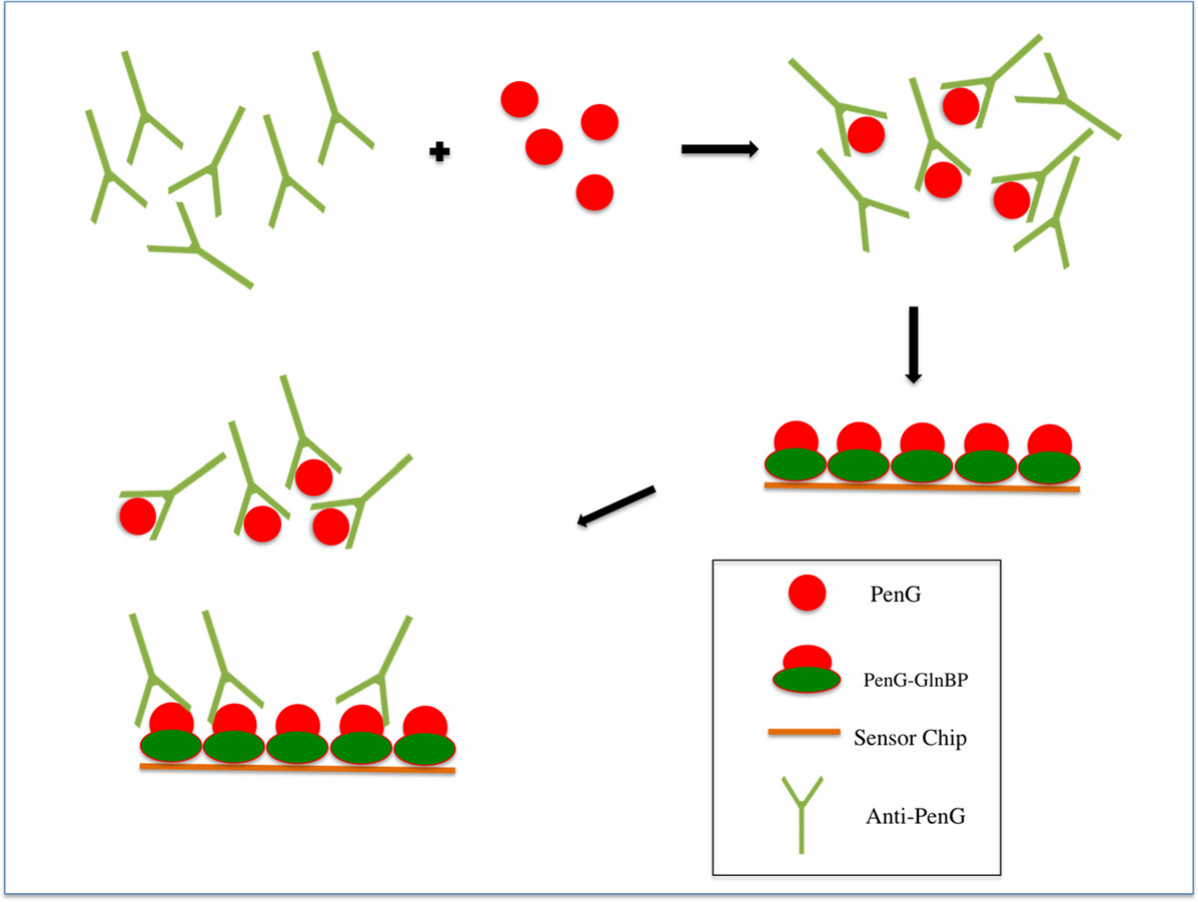
SPR-based immunoassay. Schematic representation of the competitive SPR-based immunoassay for the detection of PenG using a functionalized chip.

**Fig 4:**
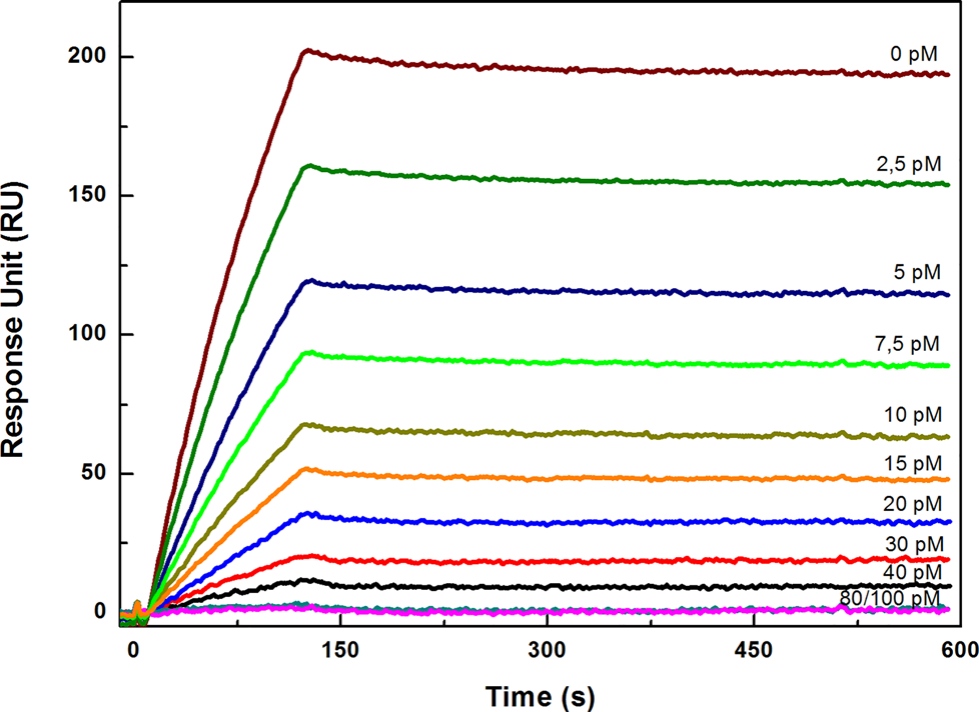
SPR competitive immunoassay measurements. Sensorgram of the competitive immunoassay. All measurements were performed three times in HBS-EP buffer at 25°C.

Following the binding experiments, a competitive immunoassay was performed. In Fig 3, the scheme describes the principles of the competition between PenG immobilized on the chip and free PenG in solution. To evaluate the potential application of our system as a competitive assay for PenG detection, different samples of antibodies at concentrations of 100 nM were pre-incubated with increasing concentrations of PenG, ranging from 0.0 pM to 100 pM. In Fig 4, the competitive assay sensorgram, which was obtained by injection of the mixture antibodies with PenG on chip surface, is shown. The results show that a decrease in the signal was registered as consequence of increasing concentrations of free PenG in solution. This effect is observed because the antibodies that have already bound to the PenG in solution do not bind to the PenG-GlnBP immobilized on the chip surface. Then, the anti-PenG antibodies compete for binding to free PenG present in solution and PenG immobilized on the chip.

**Fig 5:**
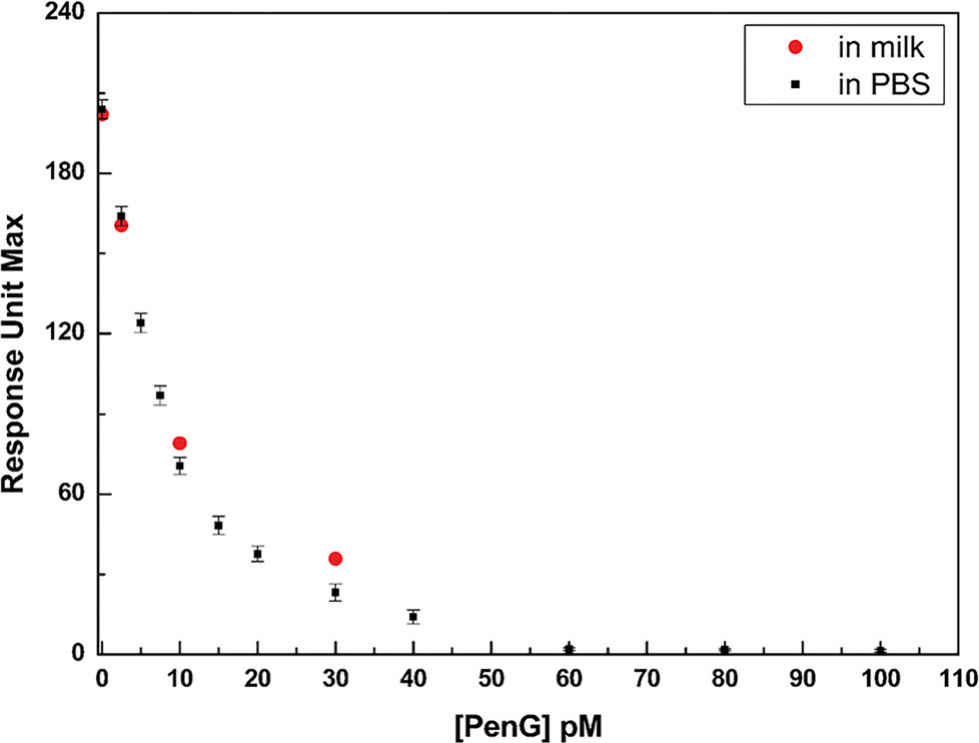
Calibration curve. Titration of the SPR-based sensing system with PenG in PBS buffer (black squares) and milk (red circles). RU_max_ values are plotted versus PenG concentration.

Fig 5 shows the dose response curve of the assay, in which the RU_max_ values from each SPR binding experiment were plotted against the PenG concentration diluted in PBS buffer and the milk solution. In analyzing the data, the limit of detection (LOD) of the assay was calculated according to Armbruster [28], and it is possible to conclude that the described method displayed a LOD of 8.0 pM.

With the aim to verify the application of the developed assay in real matrices, competitive experiments were performed diluting increased quantities of PenG directly in a milk solution. In Fig 5, the variation of the RU_max_ registered in a competitive assay performed in a milk solution diluted (1:1000) in HBS buffer is shown (red circle). The results show that in the presence of increasing concentrations of free PenG in milk, a reduction in the signal was registered. As for the laboratory samples, the reduction of the signal is a consequence of the competition for the antibody between PenG present in solution and PenG-GlnBP immobilized on the surface. Interestingly, the low concentration in milk solutions tested in this study gave exactly the same response. This result supports the possibility that the developed assay can be used directly in milk solutions without any interference from the matrices.

## Conclusions

In this work, we described the application of an easy and sensitive SPR-based method for the detection of PenG directly in a milk solution. PenG is the most common compound used in the pharmacological treatment of mastitis infection, and it is added to animal food. The illegal use and/or abuse of this compound and, in general, of β-lactam antibiotics in animal feed implies a contamination of produced milk with direct consequences to the human food chain. The obtained results note that the developed method is a promising alternative approach compared to the analytical methods that are currently used. Extensive extraction by non-trained personnel and sample clean-up are not needed. We have combined an immune-chemical approach with SPR spectroscopy to develop an efficient PenG biosensor for the detection of penicillin G outside of the laboratory. The LOD of the assay was found to be 8.0 pM, a value much lower than the MRL of the EU regulation limit that is fixed at 12 nM. Additionally, the results show the possibility to detect traces of PenG directly in milk solutions without any matrices interference. It is feasible to suggest that this would certainly be an innovative approach that would provide dairy industries with an easy-to-use, economical, rapid antibiotic test that could enable them to meet regulatory requirements and provide consumers safe and high-quality milk and dairy products.
